# Corneal antinociceptive effect of (-)-α-bisabolol

**DOI:** 10.1080/13880209.2017.1285944

**Published:** 2017-02-14

**Authors:** Gisele Façanha Diógenes Teixeira, Flávio Nogueira da Costa, Adriana Rolim Campos

**Affiliations:** a Experimental Biology Centre (Nubex), University of Fortaleza (Unifor), Ceará, Brazil;; b School of Medicine, Christus University Centre (Unichristus), Ceará, Brazil

**Keywords:** Sesquiterpene, solubilizing agents, corneal pain

## Abstract

**Context:** (-)-α-Bisabolol (BISA) is a sesquiterpene alcohol widely used as scent in cosmetic preparations, perfumes, shampoos, toilet soaps and other toiletries with potential for use in the pharmaceutical area.

**Objective:** To evaluate the corneal antinociceptive efficacy of BISA and to analyze the best solubilizing agent.

**Materials and methods:** Acute corneal nociception was induced by the local application of hypertonic saline (5 M NaCl; 20 μL) to the corneal surface of Swiss mice (*n* = 8/group) 60 min after topical treatment with solutions or ointment containing BISA (50–200 mg/mL). The number of eye wipes performed with the ipsilateral forepaw was counted for a period of 30 s. Control groups (vehicles) were included.

**Results:** BISA (50, 100 or 200 mg/mL) solubilized with Tween 80 did not reduce the number of eye wipes. Animals treated with the ointment (BISA 50, 100 or 200 mg/mL; *p* < 0.001), as well the solution containing propylene glycol (BISA 100 mg/mL; *p* < 0.05), showed significant reduction in the number of nociceptive behaviours. Solutions containing propylene glycol and isopropyl myristate had no effects.

**Discussion and conclusion:** BISA possess corneal antinociceptive activity. Although the ointment presented antinociceptive effect, it is concluded that BISA when associated with propylene glycol has better potential for corneal nociceptive pain since it is more comfortable to use, leading to greater acceptance by patients.

## Introduction

The topical drug application is preferably used in ocular disorders (Abdul Nasir et al. 2015). This form of delivery is widely used mainly because of the easiness of use and adherence to the treatment (Souza et al. 2014).

The biodistribution of drugs by this route is still a challenge, due to its low bioavailability (Abdul Nasir et al. 2013), which confers protection barriers mechanical (blinking, for example) and physiological (such as the corneal epithelium and the nasolacrimal system) that promote rapid removal of the substances from the ocular surface (Bucolo et al. 2012), which can make the absorption of the drug applied topically insufficient even when the focus of treatment is the anterior chamber (Souza et al. 2014).

The cornea has characteristics that make it an effective barrier to drug absorption: it has small size, and its relative impermeability is avascular (Das & Suresh 2010). On the corneal epithelium, the cells are lipophilic, which hinders the passage of hydrophilic substances. On the stromal layer of the cornea, there happens some difficulty of absorption of lipophilic molecules by their matrix cells consisting of hydrophilic cells (Souza et al. 2014).

For these reasons, high doses of drugs or frequent administrations are needed in order to achieve the desired result, which increases the risk of side effects (Cunha Junior et al. 2003). Aiming to have better absorption, the use of absorption facilitators (Furrer et al. 2002), microemulsions (Cunha Junior et al. 2003), liposomes (Abdul Nasir et al. 2013) or nanoparticles (Reus et al. 2009) are recommended. Systemic route can also be used, however, the drug distribution become impaired by the difficult passage through the blood-aqueous barrier and blood-retinal (Bucolo et al. 2012).

(-)-α-Bisabolol (BISA) is a sesquiterpene alcohol found in the essential oil of various plant species, among them *Matricaria chamomilla* L. and *Vanillosmopsis* are the important species (Kamatou & Viljoen 2010). This sesquiterpene is widely used as an additive in cosmetic products as anti-irritant creams, after sun lotions (Lee et al. 2010), astringent for the skin care, sunscreens and make-up (Spiegel 2012).

Recently, Solovăstru et al. (2015) reported that a spray containing ozonated oil and BISA could be a new therapeutic option for the adjuvant treatment of venous ulcers. In another study, BISA had excellent results when applied in the form of shampoo after hair transplant and sensitive scalp. This is due to the fact that it has anti-inflammatory properties and the absence of potentially irritating ingredients (Schweiger et al. 2015).

Our group has demonstrated the topical anti-inflammatory effect of BISA in experimental models of ear oedema (Leite et al. 2011) and that BISA may attenuate nociceptive sensorimotor responses and central sensitization evoked by noxious orofacial stimuli (Melo et al. 2015). Moreover, the essential oil of *Vanillosmopsis arborea* Baker rich in BISA presented antinociceptive topical effect when applied to the paw in mice and the eye (Leite et al. 2014).

This sesquiterpene is completely soluble in ethanol and isopropyl alcohol. To mix it with water, it is necessary to use solubilizing agents. This study aimed to evaluate the corneal antinociceptive efficacy of BISA and to analyze the best solubilizing agent.

## Materials and methods

### Obtaining of (-)-α-bisabolol

(-)-α-Bisabolol (purity ≥93%) was purchased from Sigma-Aldrich (St. Louis, MO).

### Animals

Eighty-four Swiss albino mice (20–30 g) from the experimental animal facility at the Christus University Center and University of Fortaleza (UNIFOR) were kept in a controlled environment (circadian cycle, 22 °C) with free access to water and standard pellet diet (Purina, São Paulo, Brazil). The experimental protocols followed the ethical guidelines of CONCEA (Brazilian Council for the Control of Animal Experimentation) and were approved by the UNIFOR Animal Research Ethics Committee under entry number 004/2012.

### Solubilizing agents

Solutions containing Tween 80 (BISA 50, 100 or 200 mg/mL), isopropyl myristate (BISA 100 mg/mL) and propylene glycol (BISA 100 mg/mL) were manufactured. Ointment [(liquid petrolatum (40%) and petrolatum (60%)] containing different concentrations of BISA (50, 100 or 200 mg/mL) was also developed.

### Eye wiping test

Corneal nociception was induced in mice by instillation of one drop (20 μL) of hypertonic saline (5 M NaCl) on the corneal surface using a fine dropper (Farazifard et al. 2005). The number of eye wipes performed with the ipsilateral forepaw during the first 30 s was registered. Mice (*n* = 6/group) were pretreated topically (20 μL) with vehicles (controls), solutions or ointment before induction.

### Statistical analysis

The results are presented as mean ± SEM of each group of six animals. The statistical analysis consisted of one-way analysis of variance (ANOVA), followed by the Tukey’s *post hoc* test for multiple comparisons. Student’s *t* test was used to determine the significance of the differences between two groups. The level of statistical significance was set at 5% (*p* < 0.05).

## Results

BISA (50–200 mg/mL) solubilized with Tween 80 did not reduce the number of eye wipes (Table 1). Animals pretreated with the ointment (at all concentrations) had a significant reduction (*p* < 0.001) in the number of nociceptive behaviour induced by hypertonic saline (Table 2). The standard concentration BISA (100 mg/mL) for the remaining experiments was determined from these results.

**Table 1.** Effect of BISA + Tween 80 on corneal nociception induced by NaCl 5M.

Group	BISA (mg/mL)	Number of eye wipes (30 s)
Control	–	12.33 ± 3.11
Tween 80 solution	50	9.91 ± 6.16
	100	10.17 ± 3.13
	200	10.58 ± 5.61

Data are expressed as mean ± SEM. ANOVA followed by Tukey test.

**Table 2.** Effect of an ointment containing BISA on corneal nociception induced by NaCl 5M.

Group	BISA (mg/mL)	Number of eye wipes (30 s)
Control	–	8.90 ± 1.03
Ointment	50	3.60 ± 0.65***
	100	4.20 ± 0.55***
	200	3.30 ± 0.55***

Data are expressed as mean ± SEM. ***p < 0.001 compared to control group. ANOVA followed by Tukey test.

BISA in solution with propylene glycol solution also presented antinociceptive activity (*p* < 0.05; Table 3). However, the solution containing isopropyl myristate presented no antinociceptive effect (Table 4).

**Table 3.** Effect of BISA + prolylene glycol in solution on corneal nociception induced by NaCl 5M.

Group	BISA (mg/mL)	Number of eye wipes (30 s)
Control	–	6.43 ± 2.12
Polypropylene glycol solution	100	0.42 ± 0.20*

Data are expressed as mean ± SEM. *p < 0.05 compared to control group. Student *t*-test.

**Table 4.** Effect of BISA + isopropyl myristate in solution on corneal nociception induced by NaCl 5M.

Group	BISA (mg/mL)	Number of eye wipes (30 s)
Control	–	6.71 ± 1.01
Isopropyl myristate	100	5.71 ± 0.55

Data are expressed as mean ± SEM. Student *t*-test.

## Discussion

The cornea is the most densely innervated tissue in the body. The majority (about 70%) of sensory afferent fibres are polymodal nociceptors activated by mechanical forces, exogenous chemical irritants, endogenously released chemical mediators and extreme temperatures (Belmonte et al. 2004). Pain management strategies include topical anaesthetics and non-steroidal anti-inflammatory with caution, since these drugs may be toxic to keratocytes (Moreira et al. 1999) and promote transient burning, stinging and conjunctival hyperaemia (Kim et al. 2010).

The development of an effective topical solution for the treatment of corneal disorders, which is able to achieve a therapeutic dose without the need for high concentrations, or frequent administration, is a challenge for biotechnology.

Analgesics currently available for the treatment of pain following ophthalmic surgery or injury are limited by transient effectiveness and undesirable or adverse side effects (Bates et al. 2010). Topical ophthalmic non-steroidal anti-inflammatory drug (NSAID) preparations have been used for a range of painful eye conditions (Smith & Goldman 2012). However, adverse events associated with ophthalmic NSAIDs include brief burning and stinging, hyperaemia of the conjunctiva, and contact dermatitis (Calder et al. 2005). A more serious complication involves the association of topical ophthalmic NSAIDs with indolent corneal ulceration and full-thickness corneal melts (Gaynes & Fiscella 2002).

Due to low toxicity, the Food and Drug Administration has classified BISA as ‘generally regarded as safe’ (GRAS), boosting its use as active ingredient in commercial products (Kamatou & Viljoen 2010). According to review published by Bhatia et al (2008), the LD_50_ for mice, *per oro*s, is 15.1 mL/kg and >5.0 g/kg for rats. Besides this, in rabbits, no changes were noted in the cornea or iris at any observation.

BISA decreased the number of eye wipes and this effect may be related 5-HT, α1, TRPV1 and central muscarinic receptors (Leite et al. 2014). Barreto et al. (2016) found the orofacial effect of BISA is associated with TNF-α but not with IL-1β. Others have reported the effect of BISA to be associated with the inhibitory activity of COX (Ortiz et al. 2016) and antioxidant effects in encephalic tissue (Leite et al. 2016). Furthermore, the antinociceptive action of BISA is not linked to a central mechanism (Rocha et al. 2011) but is more likely due to anti-inflammatory properties.

Here, some solubilizing agents have been tested in order to verify their influence on the corneal antinociceptive effect of BISA. Tween 80 surfactant is a polyethylene sorbitan ester (polysorbate), non-ionic widely used commercially to solubilize oil into water (Narayanan 2008). Its action can be limited due to their high viscosity (Shaaban & Edris 2015) and probably is not suitable for use in formulating a topical solution containing BISA.

Isopropyl myristate is an emollient ester which provides spreadability without presenting irritating or sensitizing properties (Morselli 2014). Although it shows good skin penetration (Pastore Jr & Araújo 2005), in our study, this ester abolished the antinociceptive effect of BISA.

Propylene glycol is a diol alcohol used in the pharmaceutical industry as a humectant and also in the preparation of plant extracts (Oliveira et al. 2014). It can form a film between BISA and water, increasing the elasticity and surface tension of the solution and can lead to the formation of a soluble solution, possibly allowing the antinociceptive effect of BISA (Shaaban & Edris 2015), since BISA, when in solution with propylene glycol, showed highly significant analgesic effect, reducing the nociceptive behaviour at 93.5%.

The ointment containing BISA presented antinociceptive effect at all concentrations tested. However, eye ointments have certain disadvantages as blurring of vision and sometimes have irritating effects, leading to lower acceptance by patients.

## Conclusions

BISA possesses corneal antinociceptive activity. Although the ointment presented antinociceptive effect, it is concluded that BISA when associated with propylene glycol has better potential for corneal nociceptive pain since it is more comfortable to use, leading to greater acceptance by patients.
